# Measurement properties of smartphone applications for the measurement of neck range of motion: a systematic review and meta analyses

**DOI:** 10.1186/s12891-022-05066-6

**Published:** 2022-02-10

**Authors:** E. Elgueta-Cancino, K. Rice, D. Abichandani, D. Falla

**Affiliations:** 1 Centre of Precision Rehabilitation for Spinal Pain (CPR Spine), School of Sport, Exercise and Rehabilitation Sciences, University of Birmingham, Birmingham, B15 2TT UK; 2 Division of Physiotherapy, Institute of Health and Social Care, London South Bank University, London, UK

**Keywords:** Cervical spine, Range of motion, Movement, Neck pain, Reliability, Validity

## Abstract

**Background:**

Smartphone applications offer an accessible and practical option to measure neck range of motion (ROM) and are becoming more commonly used in clinical practice. We assessed the validity, reliability, and responsiveness of smartphone applications (apps) to measure neck ROM in people with and without neck pain.

**Methods:**

A comprehensive electronic search strategy of the main electronic databases was conducted from inception until June 2021. The identified studies investigated apps which measured neck ROM, and evaluated their validity, reliability, or responsiveness, in adult participants with neck pain or asymptomatic individuals. Two independent reviewers determined eligibility and risk of bias following COSMIN guidelines. The quality of evidence was assessed according to the GRADE approach.

**Results:**

Eleven studies, with a total of 376 participants were included. Three types of apps were identified: clinometer apps, compass apps, and other apps of ‘adequate’ to ‘doubtful’ risk of bias. A meta-analysis revealed ‘good’ to ‘excellent’ intra-rater and inter-rater reliability across the three types of apps. The overall validity was rated from ‘moderate’ to ‘very high’ across all apps. The level of evidence was rated as ‘low’ to ‘very low’.

**Conclusion:**

Smartphone applications showed sufficient intra-rater reliability, inter-rater reliability, and validity to measure neck ROM in people with and without neck pain. However, the quality of evidence and the confidence in the findings are low. High-quality research with large sample sizes is needed to further provide evidence to support the measurement properties of smartphone applications for the assessment of neck ROM.

**Study registration:**

Following indications of Prisma-P guidelines, this protocol was registered in PROSPERO on 1/05/2021 with the number CRD42021239501.

**Supplementary Information:**

The online version contains supplementary material available at 10.1186/s12891-022-05066-6.

## Background

One third of the world current population is estimated to need rehabilitation, with musculoskeletal conditions being the leading cause [1–3]. Spinal pain alone is reported to be the largest contributor of global disability [1] and neck pain specifically is the fourth highest cause of years lived with disability [1, 4]; a condition associated with substantial costs [2].

The measurement of neck range of motion (ROM) is a common clinical assessment used to evaluate people with neck pain to determine the presence of functional limitations [3]. This objective measurement can be used to help identify movement impairment and can provide relevant prognostic data [4]. Additionally, ROM measures are often utilised throughout a patient’s clinical journey as objective markers to help determine progress and the effectiveness of an intervention(s) [5]. Further, measures of neck ROM are used in classification systems [3, 6, 7] and may assist in differential diagnosis [8].

There are various performance-based outcome measures (PBOM) available to measure neck ROM including measuring tapes, goniometers, the cervical range-of-motion (CROM) device or visual estimates [9]. Smartphone applications (apps), offer an accessible, low cost and practical option to measure neck ROM in clinical environments. For instance, apps such as the “compass” [10–12] and “clinometer” [10, 13] have been reported to have good reliability and validity for the measurement of frontal and sagittal cervical ROM when compared with gravitational inclinometers.

Previous systematic reviews have synthesised available evidence to determine the measurement properties of PBOM to evaluate ROM in general joint angles [14] or more specifically, for the measurement of spinal movements [15]. The main conclusions of these reviews are that there is low quality evidence supporting the measurement properties of apps to assess joint angles due to their heterogeneous nature [14] and no research has explored the responsiveness of different mobile apps. Specifically for spinal movements, available apps showed good levels of reliability and validity for neck ROM in the sagittal and frontal plane but highlighted the lack of evidence to support measurements in the horizontal plane [15].

The Consensus-based Standards for the Selection of Health Measurement Instruments (COSMIN) initiative established a taxonomy of measurement properties which covers the following domains: reliability, validity, and responsiveness [16]. As per the current evidence, it remains unclear if smartphone apps offer an optimal PBOM for clinicians to use in practice to evaluate neck ROM. This highlights a need for a systematic review of the measurement properties of smartphone apps and therefore the purpose of this systematic review is to assess the measurement properties (validity, reliability, and responsiveness) of apps to measure neck ROM in people with and without neck pain.

## Methods

This systematic review was designed using COSMIN guidelines [17] and is reported in line with The Preferred Reporting Items for the Systematic Reviews and Meta-Analysis guidelines (PRISMA) checklist [18]. The review was prospectively registered with PROSPERO (Registration CRD42021239501) on the 1st of March 2021. Ethical approval was not required since no new original data were collected given that this is a systematic review.

### Eligibility criteria

#### Inclusion criteria

Studies included in this systematic review investigated at least one measurement property of a smartphone app to measure neck ROM. The target population were adults aged 18 years or over, who were either asymptomatic or presented with neck pain as defined by International Classification of Diseasses [19]. The studies were required to have evaluated at least one of the three main domains of the COSMIN Taxonomy of measurement properties, namely, validity, reliability, and/or responsiveness [16]. Only studies written in English were included.

#### Exclusion criteria

Studies which solely investigated neck ROM using special devices such as a Cervical Range of Motion (CROM) device, goniometers or inclinometers were excluded. Conference abstracts, systematic reviews and articles without full text availability were excluded.

### Information sources

Multiple subject-specific electronic databases were systematically searched in line with Cochrane collaboration recommendations. These databases were CINAHL Plus (EBSCO interface), MEDLINE (OVID interface), SPORTDiscus (EBSCO interface) and EMBASE (OVID interface). The literature search was conducted from inception to 25th June 2021.

### Search strategy

A search strategy was formed for MEDLINE and adapted to other databases. Search terms were generated for a total of four concepts: ‘Range of motion’, ‘Neck’, ‘Measurement properties’, and ‘Smartphone application’. MESH terms were used to form more search terms so that all relevant literature was found. The search was online only, and references were found manually if needed. See Supplementary File 1 for the full MEDLINE (Ovid) search strategy. Grey literature and conference papers were searched to reduce potential publication biases.

### Study selection

Data was managed in Clarivate Analytics Endnote Version 20 Software. This allowed ease of access, duplicates to be found and removed, and the storing of full texts and abstracts. Two reviewers (KR and DA) screened the titles and abstracts of the studies using the eligibility criteria. The articles were categorized as eligible/unsure/ineligible [20]. When a study was classified as eligible the full text was screened to ensure eligibility. Studies classified as “unsure” were discussed between the two reviewers. In the event of a disagreement between the two reviewers, a third reviewer (DF) adjudicated the eligibility of the text. The number of included/excluded studies is presented with the PRISMA flow diagram with reasons for exclusions [18].

### Data extraction

A data extraction form was piloted with two articles to assess its practicality and any necessary changes were made. The form was used by the reviewers (KR and DA) independently to extract information from the eligible studies [21]. Any discrepancies were discussed and mediated by the third reviewer (DF).

### Risk of Bias

Two reviewers (KR and DA) independently assessed the risk of bias within each study using the COSMIN risk of bias checklist; this checklist was chosen due to its good level of inter-rater reliability [16]. The checklist was originally designed for use with patient reported outcome measures, but the COSMIN group have stated that the tool can be used with other types of measures including Performance-based Outcome Measures (PBOM) [17]. For instance, in the current study the factor “Internal structure” was not considered as it is not applicable to our outcome of interest. The outcome measures were scored either ‘very good’, ‘adequate’, ‘doubtful’ or ‘inadequate’ and any dispute was settled by the third reviewer (DF) [17]. The measurement properties for all of the outcomes measurements identified in the articles was assessed and summarised in table, as sufficient (+), insufficient (−), inconsistent (±), or indeterminate (?).

### Confidence in cumulative evidence

To assess the quality of pooled evidence, the Grading of Recommendations Assessment, Development, and Evaluation (GRADE) approach were adopted considering each measurement property for each type of application [22]. Four factors were examined following the COSMIN recommendation: (1) risk of bias (methodological quality of the studies), (2) inconsistency (unexplained inconsistency of results across studies), (3) imprecision (sample size of the available studies), and (4) indirectness (evidence from different populations than the population of interest in the review). ‘Publication bias’ was not examined due to the lack of registries for studies on measurement properties. The evidence was graded as either high, moderate, low or very low evidence [22].

### Data synthesis

Following the COSMIN guideline for systematic reviews [17], either a meta-analysis or narrative synthesis was conducted, based on the heterogeneity of the included studies. For a meta-analysis to be indicated, an adequate number of studies that contained similar study demographics, design and low/moderate heterogeneity were needed to be included. The I^2^ statistical analysis was used to evaluate the variation between studies that was due to heterogeneity rather than chance [23]. Heterogeneity was considered ‘substantial’ if the *I*^*2*^ scores were > 50% [23].

The meta-analysis was performed in R (version 1.4.1106). Due to the expected variability between the studies, the standard generic inverse variance random effects model was used. The correlation coefficients were converted to Fisher’s z scores and then pooled. Fisher z scores were then converted back into weighted Interclass correlation coefficients (ICCs). To summarise the results, forest plots with 95% confidence intervals were generated. For the outcomes where there was a lack of homogeneity, a narrative synthesis was conducted in line with the narrative synthesis in systematic reviews recommendation [24].

The following outcomes were included in the meta-analysis: intra-rater reliability of inclinometer apps in asymptomatic participants, intra-rater and inter-rater reliability of inclinometer apps in people with chronic neck pain, intra-rater reliability of the other apps in people with chronic neck pain. ICC indices were interpreted as follow: ICC < 0.40 was considered poor reliability, ICC = 0.40-0.58 was classed as fair reliability, ICC = 0.59-0.74 was classed as good reliability, and ICC = 0.75-1.00 was classed as excellent reliability. Pourahmadi, et al. [25] present a framework of interpreting Pearson’s correlation coefficients when they are describing validity, where a coefficient of anything < 0.3 was considered negligible, 0.3-0.5 was considered low, 0.5-0.7 as moderate, 0.7-0.9 as high, and 0.9-1 as very high.

## Results

### Study selection

**Fig. 1:**
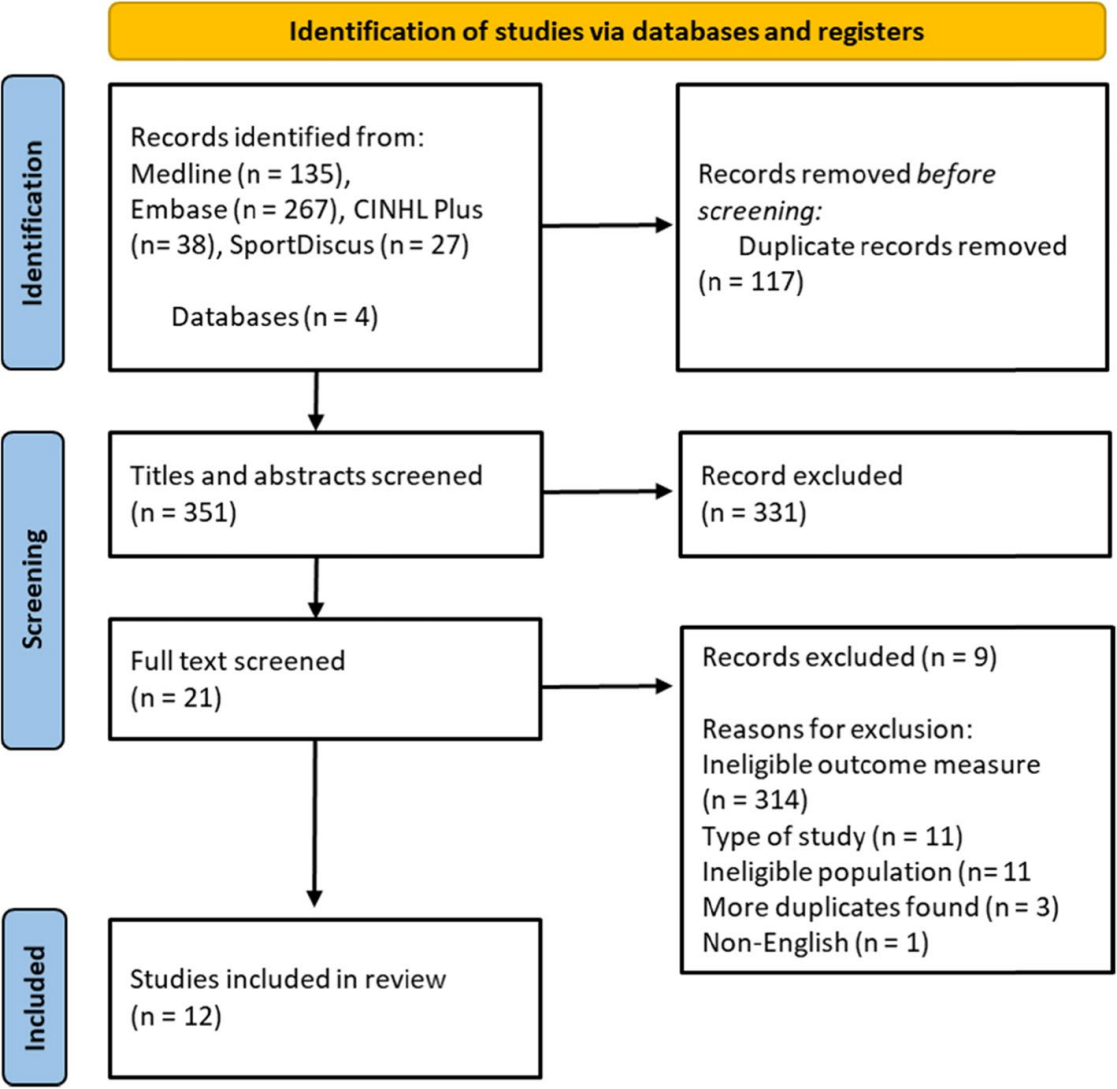
A PRISMA flow diagram

The original search identified 467 studies. After the removal of duplicates and screening of the titles and abstracts, the remaining 20 studies were subject to full-text review. Finally, 12 studies remained and were included in this systematic review. The PRISMA flow diagram is presented in Fig. 1 [18].

### Study characteristics

The 12 included studies were conducted across nine countries and had an accumulative population of 404 participants. The mean age of the participants across all the studies was 31.1 years (standard deviation 4.7 years, range 18-53 years). There were 236 females studied which is 58% of the total population. Four of the twelve studies had participants who had varied levels and durations of neck pain [10, 11, 26, 27], and eight studies had participants with no pain [12, 13, 28–33].

The smartphone apps were grouped in three types of apps, namely clinometer apps, compass apps, and other types of apps. Eleven studies assessed reliability, 7 studies assessed validity, 9 studies assessed measurement error, but no studies assessed responsiveness. A summary of the study characteristics can be found in Table 1, and further information regarding the studies including the results from each study can be found in Supplementary File 2.

**Table 1:** Study characteristics

Study	Country	Study design	Sample size	Participant characteristics Mean (SD) Mean [range] M/F	Cause of neck pain and pain characteristics	ROM tested	Smart app details
Pourahmadi et al., 2018 [26]	Iran	Cross sectional study	40	Age: 31.12 (6.38) Gender: 20 /20	Non-specific, non-traumatic neck pain Pain Intensity VAS - 3.71 ± 1.06	Neck flexion, extension, lateral flexion, and rotation	‘Goniometer Pro[G-pro]’ app (version 2.7). It works like a digital gravity-based inclinometer- iPhone 7 uses built-in accelerometer
Palsson et al., 2019 [30]	Denmark	Cross sectional study	30	Age: 2[(21-37] Gender: 19/11	Asymptomatic NA	Neck flexion, extension, and rotation	‘Balacy’ app (beta version, MEDEI, Aalborg, Denmark) custom made application
Stenneberg et al., 2018 [27]	Netherlands	Cross sectional and repeated measures design	30 for validity study, 26 for reliability study	Age: - Validity study: 53.4. Reliability study: 45.2 Gender: - Validity Study: 11/19 Reliability study: 7/19	NR NDI (median, range): Validity stud y- 9.5 (1–26) Reliability study- 11.4 (3–27) Pain duration:> 1 week	Neck flexion, extension, lateral flexion, and rotation.	‘3D range of motion’ custom made application - iPhone 4 s uses built-in gyroscope and accelerometer
Guidetti et al., 2017 [13]	Italy	Descriptive correlational study	23	Age: 26 (5) Gender:13/10	Asymptomatic NA	Neck flexion, extension, lateral flexion, and rotation.	‘Compass’ app - iPhone 5c uses built-in accelerometer, gyroscope and magnetometer
Ullucci et al., 2019 [33]	USA	NR	38	Age: 23.8 (1.2) Gender: 19/19	Asymptomatic NA	Upper cervical spine rotation	‘Clinometer’ app- Android and iPhone
Ghorbani et al., 2020 [10]	Iran	NR	20	Age: 25.9 [9-33] Gender: 7/13	NR Pain duration: > 4 weeks	Neck flexion, extension, lateral flexion and rotation.	‘Clinometer’ app - iPhone 6 s uses built-in gyroscope and accelerometer
Chang et al., 2019 [28]	Taiwan	Two stage repeated measures design	41	Age: 36.9 (7.6) Gender: 9/32	Asymptomatic NA	Neck flexion, extension, lateral flexion, and rotation.	‘GPS status & Toolbox app’ - iPhone uses built-in gyroscope and magnetometer
Satpute et al., 2019 [12]	India	Repeated measures reliability design	32	Age: 40.53 Gender: 17/15	Asymptomatic NA	Upper cervical spine rotation with FRT, and with C0-C2 test	‘Compass’ app- iPhone 6 uses built-in gyroscope, magnetometer and accelerometer
Quek et al., 2014 [31]	Australia	NR	21	Age: 31 (9.1) Gender: 11/20	Asymptomatic NA	Neck flexion, extension, lateral flexion, and rotation	Custom made app- Android phone (Samsung Galaxy S3) uses built-in accelerometer and gyroscope
Monreal et al., 2021 [29]	USA	Repeated measure, correlational design	50	Age: 21.46 (2.78) Gender: 15/35	Asymptomatic NA	Neck flexion, extension, lateral flexion and rotation	‘Clinometer’ app - Smartphone
Rodríguez-Sanza et al., 2019 [11]	Spain	Cross sectional study	Validity study: 25 Reliability study: 10	Age: - Validity study: 25 (6.72) [18-53] Reliability study: NR Gender: - Validity study: 11/14 Reliability: NR	NR Duration of neck pain: Mean (SD) (months) Validity study: 70.48 (67.25)	Upper cervical spine: flexion and extension Lower cervical spine: flexion, extension, lateral flexion and rotation	‘Clinometer’ app- Xiaomi A1 uses built-in accelerometers and a gyroscope. ‘Compass’ app-Xiaomi A1 uses built-in accelerometer.
Tousignant-Laflamme et al., 2013 [32]	Canada	Descriptive correlational design	28	Age: 23 (6) [19-43] Gender: 9/19	Asymptomatic NA	Neck flexion, extension, lateral flexion, and rotation	‘Clinometer’ app (Peter Breitling, Version 3.3)- iPhone uses built-in accelerometers ‘Compass’ app- iPhone uses built-in magnetometer.

*SD* standard deviation, *M* male, *F* female, *VAS* visual analogue scale, *NDI* neck disability index, *NR* not reported, *NA* non applicable, *FRT* flexion-rotation test

### Risk of Bias and GRADE assessment

Out of the 7 studies with participants who were asymptomatic, 3 studies scored ‘adequate’, 3 studies scored ‘doubtful’, and 1 study scored ‘very good’. Comparatively, out of the 4 studies which tested participants with neck pain, 1 study scored ‘inadequate’ and 3 scored ‘adequate’. The most common reason for the potential of risk of bias was the lack of control of the participants behaviour and the environment between repeated recordings.

For the data synthesis, results were divided by group; asymptomatic and those with neck pain. Each type of app and each measurement property (inter-rater reliability, intra-rater reliability, measurement error, criterion (concurrent) validity, and construct validity) was then considered and a summary is presented in Table 2. The overall quality of evidence (GRADE) is shown in Table 3. There was no indirectness reported in any of the measurement properties due to the subgrouping of the population into neck pain and asymptomatic groups. The characteristic of the GRADE assessments which most frequently downgraded the results was impreciseness which is based on the accumulative population for each subgroup.

**Table 2:** Summary of characteristics of the measurement properties

	Outcome measure/ measurement property					
Study	Reliability (Intra rater)	Reliability (Inter rater)	Measurement Error	Criterion Validity	Construct Validity	Responsiveness
Asymptomatic	Clinometer app. – Available free of payment in apple and google play app stores.					
Ullucci et al., 2019 [33]	✔	✔	**X**	**X**	**X**	**X**
Tousignant-Laflamme et al., 2013 [32]	✔	✔	**X**	✔	**X**	**X**
Monreal et al., 2021 [29]	✔	**X**	✔	✔	**X**	**X**
Neck pain	Goniometer – Available free of charge in apple and google play app stores.					
Pourahmadi et al., 2018 [26]	✔	✔	**X**	**X**	**X**	**X**
Ghorbani et al., 2020 [10]	✔	✔	✔	✔	**X**	**X**
Rodríguez-Sanza et al., 2019 [11]	✔	✔	**X**	✔	**X**	**X**
Asymptomatic	Compass app. – Available free of charge in apple and google play app stores.					
Guidetti et al., 2017 [13]	✔	✔	✔	✔	**X**	**X**
Satpute et al., 2019 [12]	✔	✔	✔	**X**	✔	**X**
Tousignant-Laflamme et al., 2013 [32]	✔	✔	**X**	✔	**X**	**X**
Neck pain	Compass app. – Available free of charge in apple and google play app stores					
Rodríguez-Sanza et al., 2019 [11]	✔	✔	**X**	✔	**X**	**X**
Asymptomatic	Other apps (custom made). – Non-available in app stores.					
Chang et al., 2019^a^ [28]	✔	✔	✔	**X**	✔	**X**
Quek et al., 2014 [31]	✔	**X**	✔	✔	**X**	**X**
Palsson et al., 2019 [30]	**X**	**X**	**X**	✔	**X**	**X**
Neck pain	Other apps (custom made). – Non-available in app stores.					
Stenneberg et al., 2018 [27]	✔	✔	✔	✔	**X**	**X**

^a^GPS status & Toolbox app. – Available in apple (payment required) and google play (free of charge) app stores

**Table 3:** Summary of the GRADE assessment

Outcome measure/ measurement property	Studies	Sample Size	ROB	Criteria for good Measurement Properties	ROB overall rating	GRADE - quality of evidence			
						Inconsistency	Impreciseness	Indirectness	Overall Quality of Evidence
**Asymptomatic**	**Clinometer App**								
Reliability (Intra rater)	Ullucci P.A. et al., 2019 [33]	38	D	(+)	↓	No	No	No	Moderate
	Tousignant-Laflamme et al. 2013 [32]	28	A	(−)					
	Monreal C. et al., 2021 [29]	50	A	(+)					
Reliability (Inter rater)	Ullucci P.A. et al., 2019 [33]	38	D	(+)	↓	↓	↓	No	Very Low
	Tousignant-Laflamme et al. 2013 [32]	28	A	(−)					
Measurement Error	Monreal C. et al., 2021 [29]	50	A	(?)	Serious	Inconsistent			Not rated
Criterion Validity	Tousignant-Laflamme et al. 2013 [32]	28	A	(−)	Serious	Inconsistent			Not rated
(Concurrent)	Monreal C. et al., 2021 [29]	50	D	(?)					
**Neck Pain**	**Clinometer App**								
Reliability (Intra-rater)	Pourahmadi M.R. et al., 2018 [26]	40	A	(−)	No	↓	↓	No	Low
	Ghorbani F. et al., 2020 [10]	20	A	(−)					
	Rodríguez-Sanza J. et al., 2019 [11]	10	A	(+)					
Reliability (Inter-rater)	Pourahmadi M.R. et al., 2018 [26]	40	A	(−)	No	↓		No	Low
	Ghorbani F. et al., 2020 [10]	20	A	(−)					
	Rodríguez-Sanza J. et al., 2019 [11]	10	A	(+)					
Measurement Error	Ghorbani F. et al., 2020 [10]	20	A	(?)	No	Inconsistent			Not rated
Criterion	Ghorbani F. et al., 2020 [10]	20	A	(−)	↓	↓	↓	No	Very Low
	Rodríguez-Sanza J. et al., 2019 [11]	25	I	(?)	Very serious	Inconsistent		No	Not rated
**Asymptomatic**	**Compass App**								
Reliability (Intra rater)	Guidetti. et al., 2017 [13]	23	A	(+)	↓	No	↓	No	Low
	Satpute K. et al., 2019 [12]	32	D	(+)					
	Tousignant-Laflamme et al. 2013 [32]	28	A	(−)					
Reliability (Inter rater)	Guidetti L. et al., 2017 [13]	23	A	(+)	↓	No	↓	No	Low
	Satpute K. et al., 2019 [12]	32	D	(+)					
	Tousignant-Laflamme et al. 2013 [32]	28	A	(−)					
Measurement Error	Guidetti L. et al., 2017 [13]	23	A	(?)	Serious	Inconsistent			Not rated
	Satpute K. et al., 2019 [12]	32	D	(?)					
Criterion Validity	Guidetti L. et al., 2017 [13]	23	A	(+)	No	Inconsistent			Not rated
	Tousignant-Laflamme et al. 2013 [32]	28	A	(−)					
Construct Validity (convergent)	Satpute K. et al., 2019 [12]	32	A	(?)	Serious	Inconsistent			Not rated
**Neck Pain**	**Compass app**								
Reliability (Intra rater)	Rodríguez-Sanza J. et al., 2019 [11]	10	A	(+)	↓	No	↓↓	No	Very Low
Reliability (Inter rater)	Rodríguez-Sanza J. et al., 2019 [11]	10	A	(+)	↓	No	↓↓	No	Very Low
Criterion Validity	Rodríguez-Sanza J. et al., 2019 [11]	25	I	(?)	Serious	Inconsistent			Not rated
**Asymptomatic**	**Other apps (custom-made)**								
Reliability (Intra rater)	Chang K. et al., 2019 [28]	41	D	(+)	↓↓	No	↓↓	No	Very Low
	Quek J. et al., 2014 [31]	21	D	(+)	↓ ↓	No	↓↓		Very Low
Reliability (Inter rater)	Chang K. et al., 2019 [28]	41	D	(+)	↓ ↓	No	↓↓	No	Very Low
Measurement Error	Chang K. et al., 2019 [28]	41	D	(?)	Very serious	Inconsistent			Not rated
	Quek J. et al., 2014 [31]	21	D	(?)	Very serious	Inconsistent			Not rated
Criterion Validity	Palsson T. et al., 2019 [30]	30	A	(+)	↓	No	↓↓	No	Very Low
Criterion Validity	Quek J. et al., 2014 [31]	21	A	(+)	↓	No	↓↓	No	Very Low
Construct Validity (convergent)	Chang K. et al., 2019 [28]	41	V	(+)	No	No	↓↓	No	Low
**Neck Pain**	**Other apps (custom-made)**								
Reliability (Intra rater)	Stenneberg M.S. et al., 2018 [27]	26	A	(+)	↓	No	↓↓	No	Very Low
Reliability (Inter rater)	Stenneberg M.S. et al., 2018 [27]	26	A	(+)	↓	No	↓↓	No	Very Low
Measurement Error	Stenneberg M.S. et al., 2018 [27]	26	A	(?)	Serious	Inconsistent			Not rated
Criterion Validity	Stenneberg M.S. et al., 2018 [27]	26	A	(+)	↓	No	↓↓	No	Very Low

*PBOM* Performance based outcome measure, *ROB* Risk of Bias, *GRADE* Grading of Recommendations, Assessment, Development, and Evaluations, *V* very good, *A* adequate, *D* doubtful, *I* inadequate, (+) - sufficient, (−) - insufficient, (±) - inconsistent, (?) – indeterminate, ↓ – downgraded one level

### Data synthesis

Variables that were rated as inconsistent were not graded following COSMIN guideline recommendations. No studies which assessed measurement error reported the Maximal Information Coefficient. Therefore, the measurement error was not graded.

#### Clinometer apps: asymptomatic participants

**Fig. 2:**
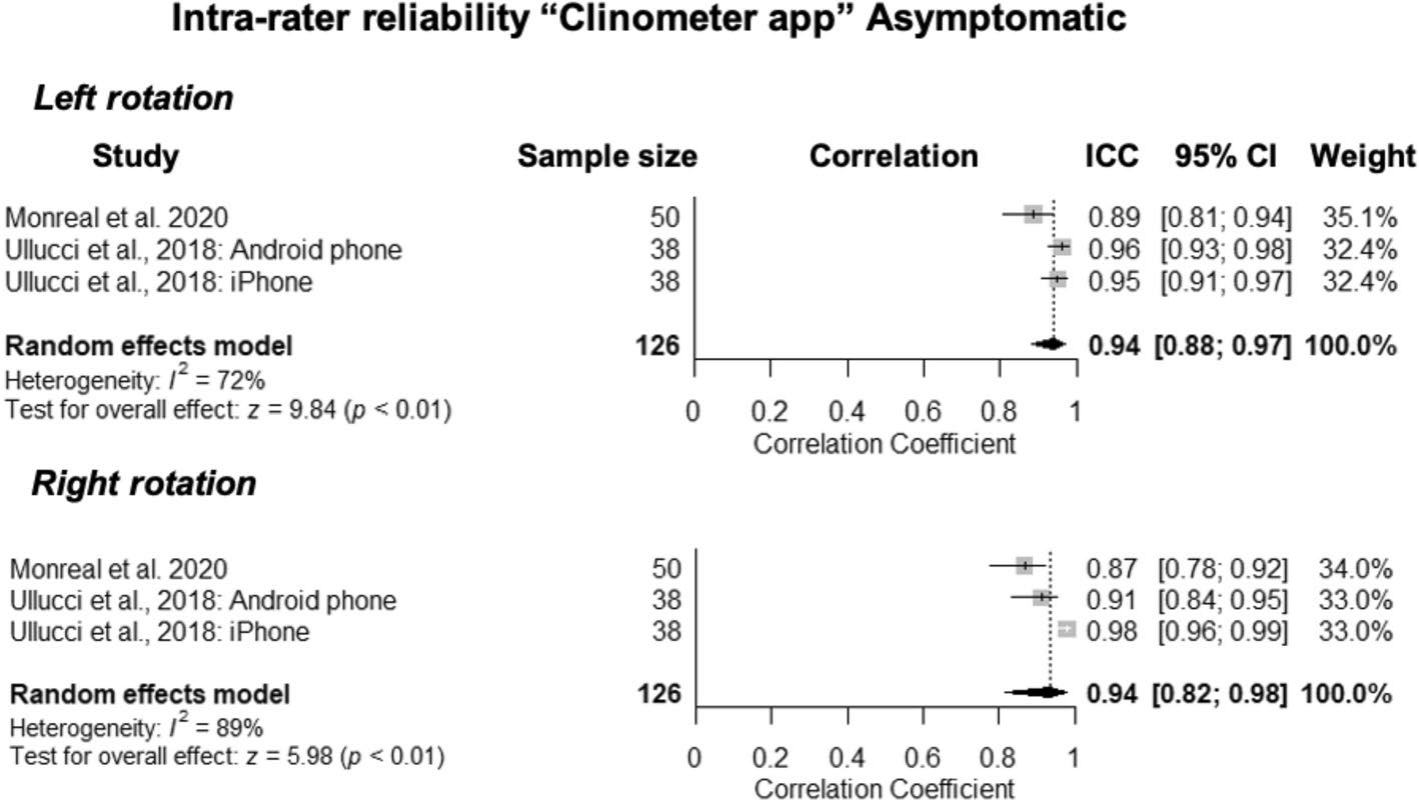
Forest plot presenting the pooled correlation coefficients for intra-rater reliability of clinometer apps for the measurement of neck range of motion in asymptomatic people. * ICC – intraclass correlation coefficient, CI – confidence interval

The rotation ROM assessment of two studies [29, 33] were pooled for intra-rater reliability meta-analyses (see Fig. 2). The weighted mean showed ICC of 0.94 (CI 95% 0.88, 0.97) for left rotation and 0.94 (CI 95% 0.82, 0.98) for right rotation, indicating excellent intra-rater reliability. The heterogeneity was deemed substantial for both movements with *I*^*2*^ scores of 72 and 89%. Ullucci et al. (2019) [33] had a risk of bias score of ‘doubtful’ and Monreal et al. (2021) [29] was rated as ‘adequate’.

Two studies assessed inter-rater reliability for clinometer apps for the measurement of neck ROM for asymptomatic participants [32, 33]. Ullucci et al. (2019) [33] reported one rater used an iPhone device and one used an Android device, meaning the ICC scores may also be influenced by these methodological differences. The inter-rater reliability of the mean total ROM produced an ICC of 0.82 (CI 95% 0.56, 0.91) and the mean peak ROM, an ICC of 0.87 (CI 95% 0.79, 0.93) which was considered ‘excellent’. While, Tousignant-Laflamme et al. (2013) [32] identified inter-rater reliability rated as ‘fair’ when measured in the sagittal and frontal planes with values that ranging from ICC = 0.40 to 0.54. The COSMIN criteria for good measurement properties indicated that clinometer apps were ‘sufficient’ for Ullucci et al. (2019) [33] and having a ‘doubtful’ risk of bias score, while Tousignant-Laflamme et al. (2013) [32] was ‘insufficient’ with an adequate risk of bias. The GRADE assessment revealed that the certainty of evidence was ‘very low’. This quality of evidence rating was downgraded by serious risk of bias, inconsistency and impreciseness due to the small population (> 50).

Two studies assessed the concurrent validity of clinometer apps in the measurement of neck ROM [29, 32] but meta- analyses could not be performed due to the heterogeneous statistical methods used. Monreal et al. (2021) [29] used Pearson’s correlation coefficient in all three planes of movement. The scores were ranged between 0.74 and 0.93, demonstrating high to very high concurrent validity. Tousignant-Laflamme et al. (2013) [32] assessed ICC revealing ‘excellent’ validity for flexion (ICC = 0.76) and right lateral flexion (ICC = 0.85), ‘good’ validity for left lateral flexion (ICC = 0.70) and ‘fair validity for extension (ICC=0.58). The GRADE assessment could not be rated given that these studies were deemed as inconsistent due to having an ‘indeterminate’ score for the COSMIN criteria for good measurement properties.

#### Clinometer apps: participants with neck pain

**Fig. 3:**
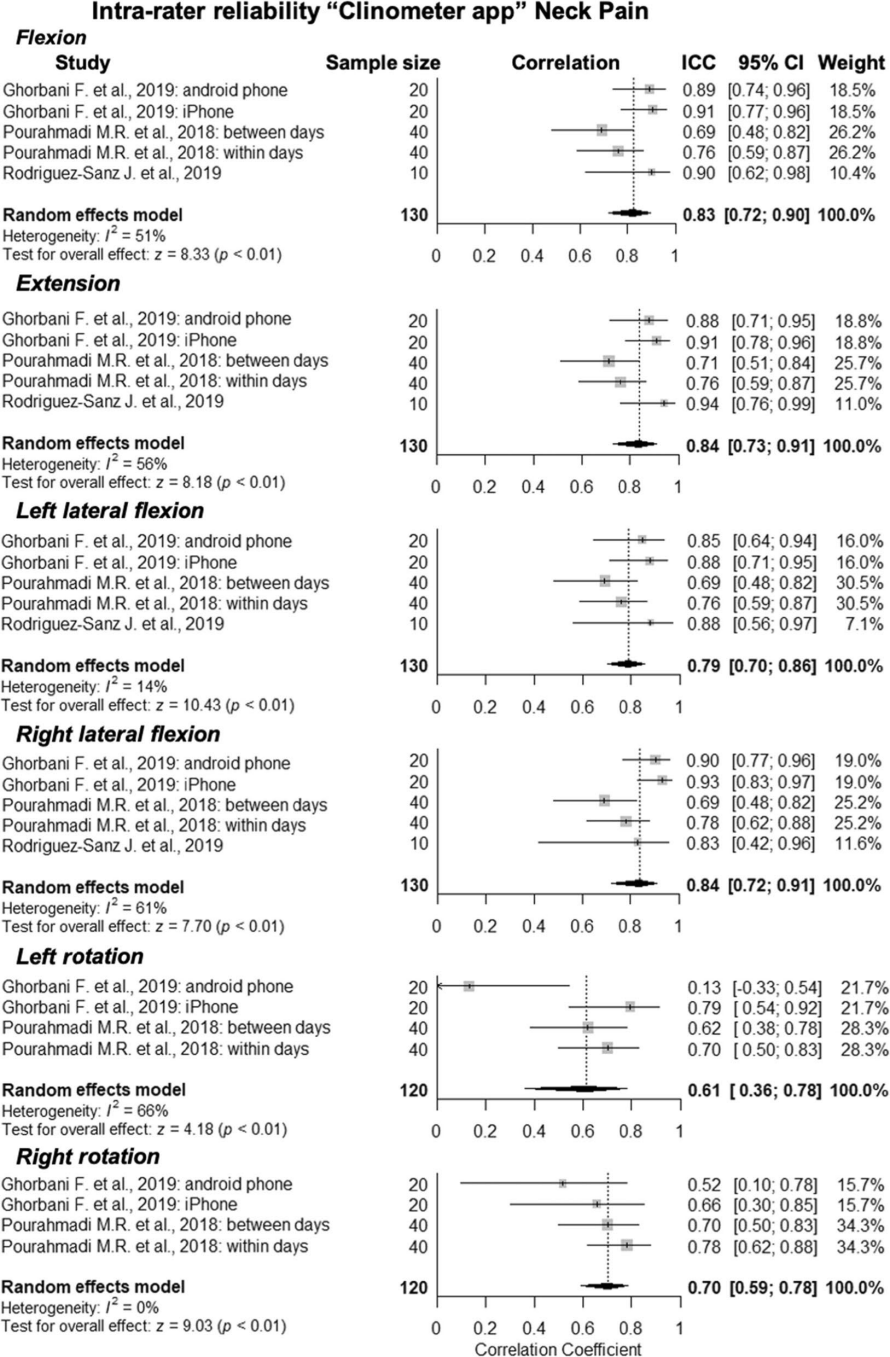
Forest plot presenting the pooled correlation coefficients for intra-rater reliability of clinometer apps for the measurement of neck range of motion in people with neck pain

Three studies assessed intra-rater reliability: Ghorbani et al. (2020) [10], Pourahmadi et al. (2018) [26], and Rodriguez-Sanz et al.(2019) [11]. However, the meta-analysis calculated weighted mean ICCs ranging between 0.61 (CI 95% 0.63, 0.78) and 0.84 (CI 95% 0.73, 0.91) for the six different movements that were measured, indicating good to excellent intra-rater reliability. Forest plots representing this data can be seen in Fig. 3. Four out of the six movements’ had substantial heterogeneity and there was a large range with *I*^*2*^ scores sitting between 0 and 66%. All studies in this subgroup were assessed as having ‘adequate’ risk of bias and the overall quality of evidence was ‘low’. This was mainly downgraded due to imprecision and inconsistency. The GRADE assessment revealed that there was ‘insufficient’ intra-rater reliability.

**Fig. 4:**
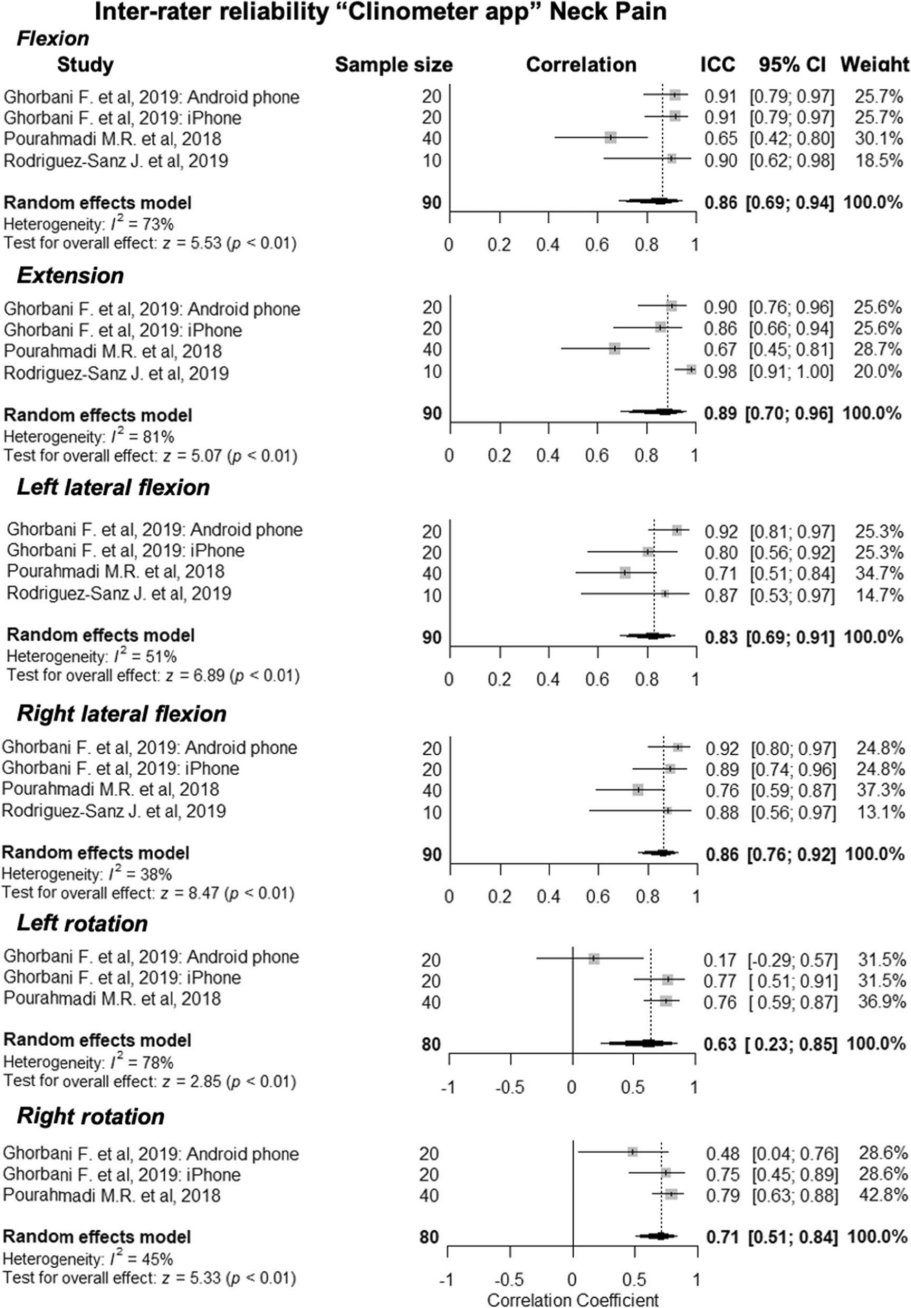
Forest plot presenting the pooled correlation coefficients for inter-rater reliability of clinometer apps for the measurement of neck range of motion in people with neck pain

Three studies evaluated inter-rater reliability [10, 11, 26]. The certainty of the evidence was graded as low quality with ‘insufficient’ inter-rater reliability for this population. Weighted mean ICCs were calculated, and the results ranged between 0.63 (CI 95% 0.23, 0.78) and 0.89 (CI 95% 0.69, 0.86) for the six different movements that were measured, indicating good to excellent intra-rater reliability. Forest plots representing this data can be seen in Fig. 4. Four out of the six measures had substantial heterogeneity and there was a large range with *I*^*2*^ scores sitting between 38 and 73%. Rodriguez-Sanz et al. (2019) [11] only used clinometer apps for movements in the sagittal and frontal plane, so this study was not included in the meta-analysis for movements in the transverse plane. All studies in this subgroup were assessed as having ‘adequate’ risk of bias and the overall quality of evidence was ‘low’. This was downgraded due to imprecision.

Two studies assessed the criterion validity of clinometer apps measuring neck ROM in people in neck pain [10, 11]. A meta-analysis could not be conducted due to the heterogeneity of the statistical methods used to measure criterion validity (ICC and Pearson’s Correlation Coefficient). Both sets of data were compared against the same “gold standard”, a Cervical Range of Motion (CROM) device. Ghorbani et al. (2020) [10] reported two sets of Pearson’s correlation coefficients from two different devices for the three planes of movement at the neck with values ranging between 0.53 and 0.94. Thus, the criterion validity was found to be moderate to very high. However, if the transverse plane movements are excluded, then the values increased to 0.72 to 0.94. Rodriguez-Sanz et al. (2019) [11] reported ICCs of between 0.92-0.98 for the measurement of neck ROM in the frontal and sagittal planes. There was ‘sufficient’ evidence to fulfil the COSMIN criteria for good measurement properties. The study by Ghorbani et al. (2020) [10] was rated as ‘adequate’ whereas the study by Rodriguez-Sanz et al. (2019) [11] was rated ‘inadequate’ for risk of bias. The overall GRADE quality of evidence for this subgroup was ‘very low’. This rating was downgraded by the inconsistency due to differences in results between the studies.

#### Compass apps: asymptomatic participants

Three studies assessed the intra-rater and inter-rater reliability of compass apps for the measurement of neck ROM in asymptomatic participants [12, 13, 32]. Satpute et al. (2019) [12] used the flexion rotation test (FRT) and upper cervical rotation (UCR) test to assess upper cervical ROM, while Guidetti et al. (2017) [13] assessed full cervical ROM in all three planes of movement and Tousignant-Laflamme, et al. (2013) [32] assessed neck ROM only in the horizontal plane of movement. It is due to these methodological differences that a meta-analysis was not indicated. Two studies reported excellent intra-rater reliability with ICC values ranging between 0.88 (CI 95%: 0.77, 0.94) [12] and 0.97 (CI 95%: 0.94, 0.99) [13] whereas Tousignant-Laflamme, et al. (2013) [32] reported ‘good’ intra-rater reliability for a first rater and ‘poor’ reliability for a second, with values of 0.74 to 0.17 respectively for measurements right and left rotation. Excellent inter-rater reliability were reported by Satpute et al. (2019) [12]   and Guidetti et al. (2017) [13] with ICC values ranging between 0.88 (CI 95%: 0.77, 0.94) and 0.99 (CI 95%: 0.98, 1.0) respectively. Tousignant-Laflamme, et al. (2013) [32] reported ‘poor’ inter-rater reliability with values ranging from 0.09 to 0.07 for right to left rotation respectively. The criteria for good measurement properties reports ‘sufficient’ intra-rater and inter-rater reliability for Satpute et al. (2019) [12] and Guidetti et al. (2017) [13]  whereas Tousignant-Laflamme, et al. (2013) [32] was ‘insufficient’. The risk of bias was rated as ‘adequate’ for Guidetti et al. (2017) [13]  and Tousignant-Laflamme, et al. (2013) [32], while Satpute et al. (2019) [12] was ‘doubtful’. The overall quality of evidence was downgraded to ‘low’ due to the risk of bias and impreciseness [12, 13].

]Criterion validity of compass apps was assessed Guidetti, et al [13]  and Tousignant-Laflamme, et al. (2013) [32]. Guidetti et al. (2017) [13]  reported ICC and Pearson’s correlation coefficient for measurements in all three planes of movement with scores ranging between 0.99 and 1 indicating ‘very high’ criterion validity. This measurement property was graded as having ‘sufficient’ criteria for good measurement properties and ‘adequate’ risk of bias. Tousignant-Laflamme, et al (2013) [32] found ‘fair’ criterion validity using for the measurement of right and left rotation (ICC: 0.55 and 0.43, respectively), ‘insufficient’ criteria for good measurement properties and ‘adequate’ risk of bias. The certainty of the evidence was downgraded to ‘low’ due to impreciseness and inconsistency.

#### Compass apps: participants with neck pain

Only one study assessed inter-rater and intra-rater reliability of compass apps in participants with neck pain [11]. The ICC value for intra-rater reliability was 0.96 (CI 95%: 0.84, 0.99) for left rotation, and 0.89 (CI 95%: 0.57, 0.97) for right rotation. The ICC values for inter-rater reliability were 0.94 (CI 95%: 0.77, 0.99) for left rotation and 0.86 (CI 95%: 0.41, 0.97) for right rotation. These scores indicate ‘excellent’ intra-rater and inter-rater reliability and were rated as being ‘sufficient’ according to the COSMIN criteria for good measurement properties. Intra and inter–rater reliability presented adequate risk of bias but the quality of the evidence was downgraded to ‘very low’ due to the small sample size which was 10 participants.

Rodriguez-Sanz et al. (2019) [11] also assessed criterion validity of a compass app for measuring neck ROM in the transverse plane. The Pearson’s correlation coefficients were 0.96 (CI 95%: 0.90, 0.99) for left rotation and 0.93 (CI 95%: 0.84, 0.97) for right rotation, suggesting that the criterion validity is ‘very high’. However, the risk of bias was ‘inadequate’, and the GRADE assessment could not rate the overall quality due to ‘indeterminate’ criteria for good measurement properties.

#### Other apps: asymptomatic participants

Chang, et al. (2019) [28] and Quek, et al. (2014) [31] evaluated intra-rater reliability of custom made apps. The criteria for good measurement properties reported ‘sufficient’ intra-rater reliability for these apps. Weighted mean ICCs ranged between 0.70 (CI 95%: − 0.61, 0.99) and 0.92 (CI 95%: 0.86, 0.95) for the six different movements that were measured, indicating ‘excellent’ intra-rater reliability. The overall quality of evidence was downgraded to ‘very low’ due to imprecision from small sample sizes and very serious risk of bias.

Chang et al. (2019) [28] assessed inter-rater reliability and the ICCs ranged between 0.88 (CI 95%: 0.78, 0.93) and 0.97 (CI 95%: 0.96, 0.98) ‘indicating excellent’ inter-rater reliability and the COSMIN criteria for good measurement properties was surpassed. The risk of bias checklist scored this study as ‘doubtful’ and the GRADE assessment rated the overall quality of evidence for this study as ‘very low’ due to the ‘very serious’ risk of bias and impreciseness.

Palsson, et al. (2019) [30] assessed criterion validity for the measurement of neck ROM in the sagittal and transverse planes. The Pearson’s correlation coefficient scores ranged between 0.82 and 0.96 which is classed as ‘high’ to ‘very high’ and so was rated as being ‘sufficient’ according to the COSMIN criteria for good measurement properties. The measurement property was graded as having ‘adequate’ risk of bias and ‘very low’ overall quality of data, downgraded due to the small sample size which was 30 participants.

Chang, et al. (2019) [28] assessed concurrent validity for the measurement of neck ROM in all planes of movement and the Pearson’s correlation coefficient ranged between 0.91 and 0.96 indicating ‘very high’ concurrent validity. This measurement property presented ‘very good’ in relation to the risk of bias with ‘low’ overall quality of evidence due to the small sample size.

#### Other apps: participants with neck pain

Stenneberg, et al. (2018) [27] assessed the inter-rater reliability of the ‘3D range of motion app’ for the measurement of neck ROM in all three planes of movement. The ICC results for intra-rater reliability were between 0.90 (CI 95%: 0.78, 0.95) and 0.96 (CI 95%: 0.09, 0.98), indicating excellent intra-rater reliability. These results were rated as being ‘sufficient’ according to the COSMIN criteria for good measurement properties. The overall quality of data according to the GRADE assessment was downgraded to ‘very low’ due to serious risk of bias and the small sample size (very serious impreciseness).

Criterion validity was assessed by Stenneberg, et al. (2018) [27] and was determined to be ‘excellent’ with ICC ranging from 0.91 (CI 95%: − 0.01, 0.98) to 0.99 (CI 95%: 0.97, 0.99). Additionally, Pourahmadi, et al. (2018) [26] demonstrated showed good to excellent concurrent validity for the measurement of the six movements assessed. Although both studies showed very low risk of bias, the certainty of the evidence was downgraded to very low due to the very serious impreciseness (small sample < 50).

## Discussion

This study systematically reviewed and synthesised the available literature that evaluated inter-rater reliability, intra-rater reliability, validity, or responsiveness of using smartphone apps to measure neck ROM in people with and without neck pain. Three types of smartphone apps were identified, namely clinometer apps, compass apps, and a group of other type of apps (e.g., costume made). From the 12 studies included in this review, no studies evaluated responsiveness, clearly indicating the need for future research to examine this measurement property of smartphone apps for the assessment of neck ROM. Overall, the three groups of apps exhibited good reliability and validity. ‘Moderate’ quality of evidence of the reliability to assess ROM in asymptomatic adults was found only for ‘Clinometer’ apps. The relative high risk of bias and small accumulative populations of the included studies, lead to ‘low’ or ‘very low’ certainty of the recommendation(s) for the other two groups of apps.

Specifically, this review found that the intra-rater and inter-rater reliability of measuring neck ROM using clinometer apps in asymptomatic people is excellent [29, 32, 33] for rotation, flexion, extension and lateral flexion, whilst when assessing people with neck pain, good to excellent intra-rater and inter-rater reliability exists [10, 11, 34]. These results support the use of clinometer apps as a reliable method for measuring neck ROM, including testing of people with neck pain. Although previous work has grouped different types of apps and did not differentiate between people with and without symptoms, similar results supporting the reliability of smartphone apps have been reported for the measurement spinal movements in the sagittal plane (flexion-extension) and frontal plane (lateral-flexion) [18]. A potential reason for the slight difference in reliability scores between those with and without neck pain is potentially due to the influence of pain on movement and a change in symptoms between assessments [35]. The studies failed to control this variable by not recording the changes in the participants’ pain between assessments. For example, one study which did not control for this was Pourahmadi, et al. (2018) [26] which, in turn, caused an increase in this study’s risk of bias score.

Intra-rater and inter-rater reliability was found to be good to excellent for the ‘other apps’. The compass apps demonstrated excellent reliability, but one [32] of the two studies only measured movement in the transverse plane. Of all movement directions, the reliability of mearing rotation was the most variable regardless of the app used or the population studies. For instance, the intra-rater reliability recorded by Quek, et al. (2014) [31] in asymptomatic participants using a custom-made app (other apps group), and by Ghorbani, et al. (2020) [10] in participants with neck pain using clinometer apps showed the lowest weighted mean ICC scores for rotation ROM. In addition, Tousignant-Laflamme, et al. (2013) [32] described poor intra-rater reliability for one of the examiners and this impacted on the inter-rater reliability described for ‘compass apps’. This may be due methodological (e.g., examiners’ experience) and technical factors (type of sensors). Specifically, magnetometers are required to measure rotation in antigravity positions, such as sitting. Magnetometers are more sensitive to signal distortion from environmental magnetic fields, therefore potentially making the measurement of rotation in these circumstances less accurate [26]. This concurs with a recent systematic review that evaluated measurement properties of apps to assess the range of spinal movement [15].

From the included studies which evaluated criterion validity, three used goniometers, one used a fluid inclinometer, three used a Cervical Range of Motion (CROM) device, two used an image-based motion capture system and one an electromagnetic tracking device. Keogh et al [36] argues that the most appropriate gold standard to use would be radiographic image-based system such as x-ray or motion capture. However, due to the financial and or ethical constraints of clinical practise, this would not always be feasible and instead, a CROM device may be the most appropriate tool for a gold standard, due to its extensively studied measurement properties [37–39]. Clinometer apps scored the least in terms of criterion validity while the ‘other apps’ group recorded the highest scores for criterion and concurrent validity.

The evidence from this review suggests that smartphone apps are a reliable and valid method of measuring neck ROM in symptomatic and asymptomatic people. This indication is in line with other research on this topic [15, 36] that shows relatively strong evidence to support the intra-rater reliability, inter-rater reliability, and validity of smartphone apps for measuring ROM in a variety of joints including the trunk. However, due to the low quality of evidence identified in this review, one specific app cannot be recommended over another for use. Future studies are required to assess responsiveness which can be achieved by evaluating changes in ROM using different apps pre and post an intervention which is known to enhance neck ROM.

### Limitations

The largest influencing factor in the low-quality ratings for risk of bias and the overall quality of evidence was the small sample size of each sub-group, especially those that examined people with neck pain. This potentially means that there is low confidence that the results represent the true measurement properties. Furthermore, fifteen out of the twenty datasets used for the meta-analyses were found to have substantial heterogeneity. This indicates a large volume of variance within the collected data which could have come from sources such as systematic errors or sampling errors. The COSMIN criteria for good measurement properties may be too simplified as if only one of the ICC scores for a study was < 0.7, then the overall result is categorised as ‘insufficient’, disregarding the other results. One specific limitation of this review is the exclusion of non-English articles.

## Conclusion

This systematic review revealed that smartphone apps may have sufficient intra-rater reliability, inter-rater reliability, and validity for the assessment of neck ROM in people with and without neck pain. Moderate quality of evidence supports the reliability of clinometer apps to assess ROM in an asymptomatic population. However, the quality of evidence of different apps when measuring people with neck pain is low, and thus there is low confidence in the findings. More high-quality research with large samples is needed to further provide evidence to support the reliability, validity, and responsiveness of smartphone apps for the assessment of neck ROM.

## Supplementary Information

**Additional file 1.**

**Additional file 2.**

## Data Availability

All data generated or analyzed data in study are included in this article. The data utilized for this article can be found individually through the articles assessed.
