# Supplementary material for: Measurement properties of smartphone applications for the measurement of neck range of motion: a systematic review and meta analyses
Source: BMC Musculoskelet Disord. 2022 Feb 10;23:138. doi: 10.1186/s12891-022-05066-6 (PMC8832814; doi:10.1186/s12891-022-05066-6)
Supplement: Supplementary file 1 — Additional file 1. [file 12891_2022_5066_MOESM1_ESM.docx]

**Supplementary File 1: Search strategy**

**MEDLINE (Ovid) Search Strategy**

1. (Validity or Construct validity or Reliability or Test-retest reliability or Responsiveness or Intrarater reliability or intra-rater reliability or intratester reliability or intra-tester reliability or repeatability or intertester reliability or inter-tester reliability or inter-rater reliability or interrater reliability or criterion validity or criterion related validity or validation or reproducible).mp. [mp=title, abstract, original title, name of substance word, subject heading word, floating sub-heading word, keyword heading word, organism supplementary concept word, protocol supplementary concept word, rare disease supplementary concept word, unique identifier, synonyms]

2. (Cervical or Neck or Head or Upper cervical or cranio-cervical or craniocervical).mp.[mp=title, abstract, original title, name of substance word, subject heading word, floating sub-heading word, keyword heading word, organism supplementary concept word, protocol supplementary concept word, rare disease supplementary word, unique identifier, synonyms]

3. (Range of motion or range of movement or movement or motion or range-of-motion or mobility).mp. [mp=title, abstract, original title, name of substance word, subject heading word, floating sub-heading word, keyword heading word, organism supplementary concept word, protocol supplementary concept word, rare disease supplementary concept word, unique identifier, synonyms]

4. (Smartphone application or iPhone application or Phone application or Cellphone application or mobile application or Smartphone app or iphone app or phone app or cellphone app or mobile app or app or application or smart phone application or smart phone app).mp. [mp=title, abstract, original title, name of substance word, subject heading word, floating sub-heading word, keyword heading word, organism supplementary concept word, protocol supplementary concept word, rare disease supplementary concept word, unique identifier, synonyms]

5. 1 and 2 and 3 and 4
