# Supplementary material for: Measurement properties of smartphone applications for the measurement of neck range of motion: a systematic review and meta analyses
Source: BMC Musculoskelet Disord. 2022 Feb 10;23:138. doi: 10.1186/s12891-022-05066-6 (PMC8832814; doi:10.1186/s12891-022-05066-6)
Supplement: Supplementary file 2 — Additional file 2. [file 12891_2022_5066_MOESM2_ESM.docx]

**Supplementary File 2: Further data extracted from the included studies**

| Study | Measurement property | Raters & testing schedule | Groups | Position/fixation of participant  Location/fixation of device | Device used in testing,  App used  Preparation or training with app  Device used as standard | Statistical measures | Results |
| --- | --- | --- | --- | --- | --- | --- | --- |
| Pourahmadi et al, 2018 | Inter-rater reliability,  intra-rater reliability,  Concurrent validity | 2 raters: Experienced PhD students physiotherapy (4-8 years)  Between day testing for intra-rater reliability  Time interval: 2 hours and 48 hours | Neck pain group | **Flexion-extension**  **Participant:** Seated ***Fixation:*** Non-elastic thoracic strap **Device:** Lateral side of the head, just beside the external auditory meatus and one axis of the app was aligned with the imaginary line between the base of the nostril and the external auditory meatus ***Fixation:*** Manually held by the rater  **Lateral flexion**  **Participant:** Seated ***Fixation:*** Non-elastic thoracic strap **Device:** The center of the app was placed over the C7 spinous process, and one axis of the app was aligned with the occipital protuberance ***Fixation:*** Manually held by the rater  **Rotation**  **Participant:** Supine ***Fixation:*** *No*  **Device:** Top of the head with the center of the app was positioned at the center of the head and one axis was aligned with the nose ***Fixation:*** Manually held by the rater  ***Other***  To standardize head posture, a visual target was adjusted to eye level in front of the participants | **Device** - iPhone 7  **App:** ‘Goniometer Pro[G-pro]’  **Experience with App** -Raters had used the G-pro app for previous research project.  **Other devices used** -universal goniometer | ICC model (3, k) for the intra-rater analysis and the ICC model (2, k) for the inter-rater analysis  Standard error of measurement (SEM), Minimum Detectable Change (95% confidence level) and Coefficient of variation (CV) was calculated  Pearson correlation coefficient (r) was calculated for concurrent validity | **Inter-rater reliability**  **ICC (2, k) (95% CI):**  Flex -- 0.65(0.35, 0.81),  Ext -- 0.67(0.38, 0.82),  L lat flex --0.71(0.39, 0.85),  R lat flex -- 0.76(0.56, 0.87),  L rot -- 0.76(0.55, 0.87),  R rot -- 0.79(0.61, 0.89)  **MDC_95_(degrees):**  Flex -- 7.70  Ext -- 7.62  L lat flex -- 5.87  R lat flex -- 4.10  L rot -- 9.14  R rot -- 9.67  **Intra-rater reliability**  **ICC (3, k) (95% CI) WITHIN DAY:**  Flx -- 0.76 (0.54, 0.87),  Ext -- 0.76 (0.54, 0.87),  L lat flx -- 0.76(0.54, 0.87)  R lat flx -- 0.78(0.58, 0.88)  L rot -- 0.70(0.43, 0.84)  R rot -- 0.78(0.59, 0.88)  **ICC (3, k) (95% CI) BETWEEN DAY:**  Flex -- 0.69 (0.42, 0.83)  Ext -- 0.71 (0.45, 0.84)  L lat flex -- 0.69 (0.42, 0.84)  R lat flex -- 0.69 (0.43, 0.84)  L rot -- 0.62 (0.30, 0.79)  R rot -- 0.70 (0.44, 0.84)  **LoA (degrees):**  Flx -- 7.95, 9.99  Ext -- 7.93, 9.41  L lat flx -- 4.04, 3.49  R lat flx -- 5.05, 5.31  L rot -- 9.35, 14.39  R rot -- 10.88, 14.40  **Concurrent validity**  **Pearson’s Correlation Coefficient:**  Flx -- 0.63  Ext -- 0.81  L lat flx -- 0.72  R lat flx -- 0.79  L rot -- 0.77  R rot -- 0.75  **SEM (degrees) WITHIN DAY:**  Flex -- 2.49  Ext -- 2.40  L lat flex -- 1.03  R lat flex -- 1.42  L rot -- 3.54  R rot -- 3.57  **SEM (degrees) BETWEEN DAY:**  Flex – 3.11  Ext – 2.57  L lat flex – 1.23  R lat flex -- 1.46  L rot – 4.51  R rot – 4.50  **MDC_95_(degrees) WITHIN DAY:**  Flex – 6.9  Ext -- 6.65  L lat flex – 2.85  R lat flex – 3.93  L rot -- 9.81  R rot -- 9.89  **MDC_95_(degrees) BETWEEN DAY:**  Flex – 8.62  Ext – 7.12  L lat flex – 3.40  R lat flex – 4.04  L rot – 12.50  R rot – 12.47 |
| Palsson et al, 2019 | Criterion validity | Not reported | Asymptomatic group | **All ROM**  **Participant:** Seated ***Fixation:*** NR **Device:** Top of the head ***Fixation:*** Holster securely fastened to a wooden plate attached to the helmet | **Device -**  iPhone 6  **App:** ‘Balacy’  **Experience with app -** No preparation reported  **Other devices used –** Motion capture system | Shapiro-Wilk test, mixed model ANOVA, Pearson’s Product moment, Bonferroni correlation, Bland-Altman plot | **Criterion Validity**  **Pearson’s correlation coefficient:**  Flx -- 0.84  Ext -- 0.82  L rot -- 0.95  R rot -- 0.96  **P – Value**  Flx -- < 0.0001  Ext -- < 0.0001  L rot -- < 0.0001  R rot -- < 0.0001  **LoA (degrees):**  Flx -- -1.2, 13.6  Ext -- -4.0, 14.3  L rot -- -2.1, 10.3  R rot -- -3.1, 10.1  **P – Value**  Flx -- < 0.0001  Ext -- < 0.0001  L rot -- < 0.0001  R rot -- < 0.0001 |
| Stenneberg et al, 2018 | Inter-rater reliability,  Intra-rater reliability  concurrent validity | 2 raters: Experienced manual therapists (10 years)  Same day testing  Time interval: 15 minutes | Neck pain group | **All ROM**  **Participant:** Seated ***Fixation:*** *No* **Device:** Forehead ***Fixation:*** Soft holder with a rigid Velcro strap around the head. | **Device -**iPhone 4s  **App:**‘3D range of motion’  **Experience with app -** No preparation reported  **Other devices used -** The Polhemus Liberty a three-dimensional electromagnetic tracking device | ICC (3,1), ICC (2,1), LoA | **Inter-rater reliability**  **ICC (2,1) (95% CI):**  Flex-Ext -- 0.90(0.78, 0.95)  Lat flex -- 0.92(0.82, 0.97)  Rot -- 0.96(0.90, 0.98)  **LoA (degrees):**  Flex/Ext -- -11.97, 15.19  Lat flex -- -10.95, 9.93  Rot-- -10.06, 13.82  **Concurrent validity**  **ICC (3,1) (95% CI):**  Flex -- 0.91(-0.01, 0.98)  Ext -- 0.99(0.97, 0.99)  L rot -- 0.95(0.04, 0.99)  R rot -- 0.91(-0.01, 0.98)  L lat flex -- 0.99(0.97, 0.99)  R lat flex -- 0.98(0.95, 0.99)  **LoA (degrees):**  Flx-- -0.21, 6.35  Ext-- -2.56, 4.15  L lat flx -- -2.11, 3.61  R lat flx -- -1.85, 3.43  L rot -- 0.60, 7.61  R rot -- 0.58, 8.08 |
| Guidetti et al, 2016 | Intra-rater reliability,  Inter-rater reliability,  criterion validity | 2 raters: Experience not declared  Measurement time intervals not reported | Asymptomatic group | **Flexion-extension, lateral flexion**  **Participant:** Seated ***Fixation:*** Shoulder strap **Device:** Top of the head ***Fixation:*** Fixed to a helmet  **Rotation**  **Participant:** Supine ***Fixation:*** Shoulder strap **Device:** Rear side of the head ***Fixation:*** Fixed to a helmet | **Device -**iPhone 5c  **App:** ‘Compass’  **Experience with app -** No preparation reported  **Other devices used -** Fluid inclinometer | ICC (3, k), ICC (2, k), OLP regression, Bland and Altman plot, LoA, SEM, MDC, Pearson’s correlation coefficient | **Intra-rater reliability**  **ICC (3, k) (95% CI):**  Flx -- 0.946(0.880, 0.977)  Ext -- 0.973(0.937, 0.988)  L lat flx -- 0.923(0.829, 0.967)  R lat flx -- 0.942(0.855, 0.976)  L rot -- 0.977(0.947, 0.990)  R rot -- 0.953(0.828, 0.983)  **% Error:**  Flx -- 13  Ext -- 11  L lat flx -- 16  R lat flx – 14  L rot -- 6  R rot -- 8  **SEM (degrees):**  Flx -- 2.54  Ext -- 2.69  L lat flx -- 2.44  R lat flx -- 2.30  L rot -- 1.62  R rot -- 1.94  **MDC_95_ (degrees):**  Flx -- 7  Ext -- 7  R lat flx -- 6  L lat flx -- 7  R rot -- 5  L rot -- 4  **LoA:**  Flx -- -6.51, 8.16  Ext -- -7.55, 8.08  R lat flx -- -4.97, 8.01  L lat flx -- -6.86, 7.82  R rot -- -3.57, 7.40  L rot -- -4.21, 4.99  **Inter-rater reliability**  **ICC (2,k) (95% CI):**  Flx -- 0.982(0.959, 0.993)  Ext -- 0.992(0.981, 0.996)  R lat flx -- 0.991(0.97, 0.996)  L lat flx -- 0.985(0.964, 0.993)  R rot -- 0.989(0.975, 0.995)  L rot -- 0.989(0.975, 0.995)  **% Error:**  Flx -- 1  Ext -- 8  R lat flx -- 8  L lat flx -- 9  R rot -- 6  L rot -- 6  **SEM (degrees):**  Flx -- 2.01  Ext -- 2.03  R lat flx -- 1.39  L lat flx -- 1.55  R rot -- 1.48  L rot -- 1.55  **MDC_95_ (degrees):**  Flx -- 6  Ext -- 6  R lat flx -- 4  L lat flx -- 4  R rot -- 4  L rot -- 4  **LoA:**  Flx -- -5.19, 6.28  Ext -- -6.57, 4.96  R lat flx -- -4.08, 3.78  L lat flx -- -5.16, 3.07  R rot -- -3.85, 4.67  L rot -- -4.65, 4.47  **Criterion validity**  **ICC (2,k) (95% CI):**  Flx -- 1(0.999, 1)  Ext -- 1(1, 1)  R lat flx -- 0.999(0.998, 1)  L lat flx -- 0.998(0.996, 0.999)  R rot -- 0.999(0.996, 0.999)  L rot -- 0.999(0.997, 0.999)  **Pearson’s correlation coefficient:**  Flx -- 0.99  Ext -- 1  R lat flx -- 0.99  L lat flx -- 0.99  R rot -- 0.99  L rot -- 0.99  **% Error:**  Flx -- 1  Ext -- 1  R lat flx -- 2  L lat flx -- 3  R rot -- 2  L rot -- 2  **SEM (degrees):**  Flx -- 0.26  Ext -- 0.28  R lat flx -- 0.38  L lat flx -- 0.52  R rot -- 0.49  L rot -- 0.49  **LoA:**  Flx -- -0.78, 0.67  Ext -- -0.99, 0.64  R lat flx -- -1.36, 0.79  L lat flx -- -1.80, 1.15  R rot -- -1.6, 0.82  L rot -- -1.74, 1.05 |
| Ullucci et al, 2018 | Inter-rater reliability and  intra-rater reliability. | 2 raters: Experienced clinical researchers (40+ years combined)  Same day testing  Time interval: none | Asymptomatic group | **All ROM**  **Participant:** Seated ***Fixation:***  NR **Device:** Forehead ***Fixation:*** Rigid holder strapped around the head. | **Devices -**Android phone and iPhone  **App:** ‘Clinometer’  **Experience with app –** no preparations reported  **Other devices used -** none | ICC was used for all measurement properties | **Inter-rater reliability:**  **ICC (95% CI):**  Mean total ROM -- 0.817(0.575, 0.914)  Mean peak ROM -- 0.872(0.786, 0.928)  **P – Value:**  Mean total ROM -- < 0.001  Mean peak ROM -- < 0.001  **Intra-rater reliability:**  **ICC (95% CI):**  Android L rot -- 0.962(0.936, 0.979)  Android R rot -- 0.912(0.849, 0.951)  iPhone L rot -- 0.951(0.917, 0.973)  iPhone R rot -- 0.979(0.964, 0.988)  **P – Value:**  Android L rot -- < 0.001  Android R rot -- < 0.001  iPhone L rot -- < 0.001  iPhone R rot -- < 0.001 |
| Ghorbani et al, 2019 | Intra-rater reliability, inter-rater reliability, criterion validity | 2 raters: Experienced PhD students (non-specified number of years)  Between day testing for intra-rater reliability  Time interval: 7 days | Neck pain group | **Flexion-extension**  **Participant:** Seated ***Fixation:*** No **Device:** Non-specified –lateral side of the head ***Fixation:*** Manually held by the rater  **Lateral flexion**  **Participant:** Seated **Fixation:** No **Device:** Rear side of the head. **Fixation:** Manually held by the rater  **Rotation**  **Participant:** Seated ***Fixation:*** No **Device:** Top of the head ***Fixation:*** Manually held by the rater | **Devices -**Android phone and iPhone  **App:** ‘Clinometer’  **Experience with app -** no preparation reported  **Other devices used** **-**  CROM device, JTech Dueler IQ Digital inclinometer | Shapiro Wilk test, ICC (3,k), ICC (2,k), SEM, MDC, Pearson’s correlation coefficient, Bland-Altman plot, LoA values. | **Intra-rater reliability**  **iPhone ICC (3, k) (95% CI):**  Flx -- 0.906(0.763, 0.964)  Ext -- 0.91(0.763, 0.964)  R lat flx -- 0.93(0.825, 0.974)  L lat flx -- 0.878(0.673, 0.95)  R rot -- 0.656(0.113, 0.873),  L rot -- 0.794(0.449, 0.915)  **Android ICC (3, k) (95% CI):**  Flx -- 0.89(0.708, 0.955),  Ext -- 0.879(0.698, 0.956)  R lat flx -- 0.903(0.756, 0.963)  L lat flx -- 0.846(0.613, 0.942)  R rot -- 0.517(-0.27, 0.821)  L rot -- 0.131(-1.423, 0.677)  **Inter-rater reliability**  **iPhone ICC (2, k) (95% CI):**  Flx -- 0.914(0.788, 0.966)  Ext -- 0.855(0.644, 0.944)  R lat flx -- 0.89(0.731, 0.958)  L lat flx -- 0.803(0.444, 0.912)  R rot -- 0.746(0.346, 0.892)  L rot -- 0.775(0.417, 0.905)  **Android ICC (2, k) (95% CI):**  Flx -- 0.912(0.724, 0.961)  Ext -- 0.902(0.761, 0.962)  R lat flx -- 0.919(0.79, 0.966)  L lat flx -- 0.92(0.805, 0.969)  R rot -- 0.477(-0.345, 0.801)  L rot -- 0.174(-0.984, 0.663)  **Criterion validity**  **iPhone Pearson’s correlation coefficient:**  Flx -- 0.866  Ext -- 0.83  R lat flx -- 0.942  L lat flx -- 0.871  R rot -- 0.694  L rot -- 0.638  **P-Value:**  Flx -- <0.001  Ext -- <0.001  R lat flx -- <0.001  L lat flx -- <0.001  R rot -- 0.001  L rot -- 0.002  **Android Pearson’s correlation coefficient:**  Flx -- 0.725  Ext -- 0.875  R lat flx -- 0.915  L lat flx -- 0.91  R rot -- 0.535  L rot -- 0.458  **P-Value:**  Flx -- <0.001  Ext -- <0.001  R lat flx -- <0.001  L lat flx -- <0.001  R rot -- 0.015  L rot -- 0.042  **Measurement Error:**  **iPhone SEM (degrees):**  Flx -- 3.263  Ext -- 3.297  R lat flx -- 1.908  L lat flx -- 2.415  R rot -- 3.52  L rot -- 2.937  **iPhone MDC (degrees):**  Flx -- 9.041  Ext -- 9.135  R lat flx -- 5.287  L lat flx -- 6.691  R rot -- 9.753  L rot -- 8.138  **Android SEM (degrees):**  Flx -- 3.331  Ext -- 4.386  R lat flx -- 2.488  L lat flx -- 2.489  R rot -- 8.678  L rot -- 11.864  **Android MDC (degrees):**  Flx -- 9.23  Ext -- 12.153  R lat flx -- 6.894  L lat flx -- 6.897  R rot -- 24.046  L rot -- 32.875 |
| Chang et al, 2019 | Intra-rater reliability, inter-rater reliability, construct validity | 2 raters: Physiotherapists (more than 1 year)  Same day testing | Asymptomatic group | **All ROM**  **Participant:** Seated ***Fixation:***  No **Device:** Forehead ***Fixation:*** Placed on virtual reality goggles which were strapped to the patient’s face | **Device –**iPhone  **App:** ‘GPS status & Toolbox app’  **Experience with app -**  No preparation reported  **Other devices used -** universal goniometer | ICC, SEM, MDC, Bland-Altman plot, LoA, paired t-test, Pearson’s correlation coefficient. | **Intra-rater reliability**  **ICC (95% CI):**  Flx -- 0.963(0.933, 0.980)  Ext -- 0.954(0.916, 0.975),  R lat flx -- 0.925(0.864, 0.959),  L lat flx -- 0.939(0.888, 0.967)  R rot -- 0.959(0.925, 0.978)  L rot -- 0.931(0.874, 0.962)  **MDC (degrees):**  Flx -- 7.42  Ext -- 6.92  R lat flx -- 4.44  L lat flx -- 4.12  R rot -- 4.61  L rot -- 5.52  **SEM (degrees):**  Flx -- 2.68  Ext -- 2.50  R lat flx -- 1.60  L lat flx -- 1.48  R rot -- 1.66  L rot -- 1.99  **Inter-rater reliability**  **ICC (95% CI):**  Flx -- 0.979(0.962, 0.989)  Ext -- 0.953(0.914, 0.975)  R lat flx -- 0.880(0.786, 0.934)  L lat flx -- 0.931(0.875, 0.963)  R rot -- 0.942(0.895, 0.969)  L rot -- 0.956(0.919, 0.976)  **MDC (degrees):**  Flx -- 5.01  Ext -- 7.24  R lat flx -- 5.63  L lat flx -- 4.39  R rot -- 5.44  L rot -- 4.51  **SEM (degrees):**  Flx -- 1.81  Ext -- 2.61  R lat flx -- 2.03  L lat flx -- 1.59  R rot -- 1.96  L rot -- 1.63  **Construct validity**  **Pearson’s correlation coefficient (95% CI):**  Flx -- 0.968(0.941, 0.983)  Ext -- 0.969(0.944, 0.984)  R lat flx -- 0.941(0.892, 0.968)  L lat flx -- 0.918(0.852, 0.956)  R rot -- 0.927(0.867, 0.961)  L rot -- 0.921(0.857, 0.957) |
| Satpute et al, 2018 | Inter-rater reliability, intra-rater reliability. | 2 raters: 1.Experienced physiotherapist (15 years). 2. Inexperienced physiotherapist (undergraduate intern)  Between day testing for intra-rater reliability  Time interval: 24 hours | Asymptomatic group | **Flexion-rotation test**  **Participant:** Supine ***Fixation:***  No **Device:** Top of the head ***Fixation:*** Strapped to the head with Velcro  .  **C0-C2 test in sitting**.  **Participant:** Seated ***Fixation:***  No **Device:** Top of the head ***Fixation:*** Strapped to the head with Velcro | **Device –**iPhone  **App:** ‘Compass’  **Experience with app -**Examiner had extensive training for measurement procedure  **Other devices used** **-** none | Independent samples t-test, ICC (2,1), SEM, MDC, Pearson’s correlation (between the two tests), simple linear regression analysis | **Inter-rater reliability**  **ICC (2, 1) (95% CI):**  FRT R rot -- 0.93(0.85, 0.97)  FRT L rot -- 0.92(0.84, 0.96)  UCR R rot -- 0.92(0.84, 0.96)  UCR L rot -- 0.89(0.78, 0.94)  **MDC_90_ (degrees):**  FRT R rot -- 4.05  FRT L rot -- 3.98  UCR R rot -- 1.77  UCR L rot -- 2.27  **SEM (degrees):**  FRT R rot -- 1.74  FRT L rot -- 1.71  UCR R rot -- 0.76  UCR L rot -- 0.97  **Intra-rater reliability**  **ICC (2, 1) (95% CI):**  FRT R rot -- 0.90(0.80, 0.95)  FRT L rot -- 0.91(0.82, 0.95)  UCR R rot -- 0.91(0.82 0.96)  UCR L rot -- 0.88(0.77, 0.94)  **MDC_90_ (degrees):**  FRT R rot -- 4.85  FRT L rot -- 4.23  UCR R rot -- 1.95  UCR L rot -- 2.38  **SEM (degrees):**  FRT R rot -- 2.08  FRT L rot -- 1.81  UCR R rot -- 0.84  UCR L rot -- 1.02  **Validity**  **Pearson’s correlation coefficient comparing the two tests:**  L rot -- 0.84  R rot -- 0.83 |
| Quek et al, 2014 | Intra-rater reliability, concurrent validity | 1 rater: Experienced physiotherapist (12 years)  Between day testing for intra-rater reliability  Time interval: 1-7 days | Asymptomatic group | **All ROM**  **Participant:** Seated ***Fixation:*** Shoulder strap  **Device:** Top of the head ***Fixation:*** Fixed to a helmet | **Device -** Samsung Galaxy S3  **App:** Custom made app  **Experience with app -** No preparation reported  **Other devices used -**  9 camera motion analysis system | Spearman’s correlation coefficient, ICC, Bland and Altman plot, LoA, OLP regression, SEM, MDC. | **Intra-rater reliability**  **ICC (3,3) (95% CI):**  Flx -- 0.86(0.38, 0.96)  Ext -- 0.82(0.49, 0.94)  R lat flx -- 0.90(0.73, 0.97)  L lat flx -- 0.85(0.57, 0.95)  R rot -- 0.33(−0.34, 0.73)  L rot -- 0.05(−1.7, 0.67)  **MDC (degrees):**  Flx -- 9.2  Ext -- 11.9  R lat flx -- 8.3  L lat flx -- 12.2  R rot -- 48.7  L rot -- 46.9  **SEM (degrees):**  Flx -- 3.1  Ext -- 5.0  R lat flx -- 2.8  L lat flx -- 4.1  R rot -- 16.4  L rot -- 15.8  **Width of 95% LOA:**  Flx -- 9.1  Ext -- 11.8  R lat flx -- 8.3  L lat flx -- 11.8  R rot -- 48.2  L rot -- 46.8  **% Error:**  Flx -- 9  Ext -- 7  R lat flx -- 9  L lat flx -- 12  R rot -- 40  L rot -- 35  **Concurrent validity**  **Spearman’s correlation coefficient (95% CI):**  Flx -- 0.99 (0.30, 0.996)  Ext -- 0.83 (0.80, 0.97)  R lat flx -- 0.93 (0.71, 0.99)  L lat flx -- 0.92 (0.89, 0.98)  R rot -- 0.81 (-0.13, 0.85)  L rot -- 0.52 (-0.60, 0.80)  **ICC (3, 3):**  Flx -- 0.98  Ext -- 0.92  R lat flx -- 0.96  L lat flx -- 0.95  R rot -- 0.53  L rot -- 0.53  **Width of 95% LOA:**  Flx -- 2.3  Ext -- 9.6  R lat flx -- 4.6  L lat flx -- 7.1  R rot -- 9.6  L rot -- 18.6  **% Error:**  Flx – 2  Ext -- 6  R lat flx -- 5  L lat flx -- 7  R rot -- 8  L rot – 14 |
| Monreal et al, 2020 | Intra-rater reliability, concurrent validity. | 1 rater: Experienced athletic training (7 years)  Same day testing | Asymptomatic group | **Flexion-extension: Participant:** Seated **Fixation:** Non-elastic thoracic strap **Device:** on the left side of the head, aligned with external auditory meatus  **Fixation:** Manually held by the rater  **Lateral flexion Participant:** Seated **Fixation:** Non-elastic thoracic strap **Device:** Lateral side of the head. On the contralateral side, with the display level aligned with the participant’s eyes **Fixation:** Manually held by the rater  **Rotation Participant:** Supine  **Device:** Top of the head with the display arrow aligning with the nose and the display interface bar parallel to an imaginary line between the acromial processes **Fixation:** Manually held by the rater | **Device -**Smartphone  **App:** ‘Clinometer’  **Experience with app -**Minimal experience with clin-app but experience with goniometry and inclinometry expected of athletic trainer  **Other devices used -** baseline goniometer. | Pearson’s correlation coefficient, ICC (3,1), SEM, MDC | **Intra-rater reliability**  **ICC (3,1):**  Flx -- 0.725  Ext -- 0.799  L lat flx -- 0.930  R lat flx -- 0.923  L rot -- 0.888  R rot -- 0.868  **Concurrent validity**  **Pearson’s correlation coefficient:**  Flx -- 0.743  Ext -- 0.842  L lat flx -- 0.930  R lat flx -- 0.932  L rot -- 0.890  R rot -- 0.916  **Initial measurements MDC (degrees):**  Flx -- 1.89  Ext -- 2.16  L lat flx -- 2.47  R lat flx -- 2.45  L rot -- 1.57  R rot -- 1.43  **Repeat measurements MDC (degrees):**  Flx -- 2.05  Ext -- 2.39  L lat flx -- 2.42  R lat flx -- 2.48  L rot -- 1.56  R rot -- 1.43 |
| Rodríguez-Sanza et al, 2019 | Inter-rater reliability,  intra-rater reliability,  criterion validity. | 2 raters: Physiotherapists  Same day testing | Neck pain group | **Upper cervical spine *flexion-extension:***  **Position:** Standing with posterior head against wall  **Fixation:** Manually stabilized thoracic spine and shoulder  **Device:** Lateral side of the head, aligned with the ear  **Fixation:** Manually held by the rater  **Lower cervical spine movements: *flexion-extension***  **Position:** Seated  **Fixation:** No  **Device:** Lateral side of the head, aligned with the ear  **Fixation:** Manually held by the rater  ***Lateral flexion***  **Position:** Seated  **Fixation:** No  **Device:** Lateral side of the head, contralateral side aligned with the eyes  **Fixation:** Manually held by the rater  ***Rotation***  **Participant:** Seated  **Fixation:** No  **Device:** Top of the head with the device aligning with the nose.  **Fixation:** Manually held by the rater | **Device -**Xiaomi A1 smartphone  **App:** ‘Clinometer’& ‘Compass’  **Experience of app -**Examiners practiced on 15 other subjects prior to study  **Other devices used** **-**  CROM device | ICC was used for all measurement properties | **Intra-rater reliability**  **ICC (95% CI):**  UCS flx -- 0.73(–0.07, 0.93)  UCS ext -- 0.98(0.93, 1)  LCS flx -- 0.90(0.60, 0.98)  LCS ext -- 0.94(0.70, 0.99)  LCS R lat flx -- 0.83(0.34, 0.96)  LCS L lat flx -- 0.88(0.23, 0.97)  LCS R rot -- 0.89(0.57, 0.97)  LCS L rot -- 0.96(0.84, 0.99)  **Inter-rater reliability**  **ICC (95% CI):**  UCS flx -- 0.68 (-0.32, 0.92)  UCS ext -- 0.97 (0.90, 0.99)  LCS flx -- 0.90 (0.56, 0.98)  LCS ext -- 0.98 (0.92, 1)  LCS R lat flx -- 0.88 (0.52, 0.97)  LCS L lat flx -- 0.87 (0.30, 0.97)  LCS R rot -- 0.86 (0.41, 0.97)  LCS L rot -- 0.94 (0.77, 0.99)  **Concurrent validity**  **ICC (95% CI):**  UCS flx -- 0.98(0.95, 0.99)  UCS ext -- 0.97(0.94, 0.99)  LCS flx -- 0.98(0.95, 0.99)  LCS ext -- 0.98(0.97, 1)  LCS R lat flx -- 0.92(0.81, 0.97)  LCS L lat flx -- 0.96(0.90, 0.98)  LCS R rot -- 0.93(0.84, 0.97)  LCS L rot -- 0.96(0.90, 0.99) |
| Tousignant-Laflamme et al, 2013 | Inter-rater reliability, intra-rater reliability, criterion validity. | 2 raters: Physiotherapy students  Same day testing | Asymptomatic group | **Flexion-extension**  **Position:** Seated  **Fixation:** Thorax strap  **Device:** Lateral side of the head, aligned with the ear insertion to the head  **Fixation:** Manually held by the rater  **Lateral flexion**  **Position:** Seated  **Fixation:** Thorax strap  **Device:** Lateral side of the head, contralateral side aligned with the corner of the eye  **Fixation:** Manually held by the rater  ***Rotation***  **Participant:** Seated  **Fixation:** Thorax strap  **Device:** Top of the head with the compass aligned in front with the nose  **Fixation:** Manually held by the rater | **Device –**Smartphone  **App:** ‘Clinometer’ & ‘Compass’  **Experience with app** – one hour  **Other devices used -** CROM | ICC was used for all measurement properties | **Intra-rater reliability**  **ICC (95% CI):**  **Rater 1**  Flx -- 0,78(0,58-0,89)  Ext -- 0,84(0,69-0,92)  R lat flx -- 0,77(0,56-0,89)  L lat flx -- 0,78(0,59-0,89)  R rot -- 0,74(0,51-0,87)  L rot -- 0,66(0,39-0,83)  **Rater 2**Flx -- 0,68(0,41-0,84)  Ext -- 0,42(0,06-0,68)  R lat flx -- 0,68(0,42-0,83)  L lat flx -- 0,68(0,41-0,84)  R rot -- 0,17( −0,21-0,5)  L rot -- 0,28(−0,54-0,67)  **Inter-rater reliability**  **ICC (95% CI):**  Flx -- 0,48(0,14-0,72)  Ext -- 0,49 0,15-0,72  R lat flx -- 0,54(0,22-0,75)  L lat flx -- 0,40(0,04-0,67)  R rot -- 0,09(−0,28-0,44)  L rot -- 0,07(−0,30-0,42)  **Criterion Validity**  **ICC (95% CI):**  Flx -- 0.76(0.55-0.88)  Ext -- 0.58 (0.27-0.78)  R lat flx -- 0.85(0.70-0.93)  L lat flx -- 0.70(0.46-0.85)  R rot -- 0.55(0.23-0.76)  L rot --0.43(0.08-0.69) |

ROM – Range of motion, ICC – Intraclass correlation coefficient, flx – flexion, ext – extension, R lat flx – right lateral flexion, L lat flx – left lateral flexion, R rot – right rotation, L rot – left rotation, MDC – minimal detectable change, FRT – flexion rotation test, UCR – upper cervical rotation, UCS – upper cervical spine, LCS – lower cervical spine, LoA – Limits of agreement, ANOVA – analysis of variance, OLP – Ordinary least products.
